# Diagnosis and treatment of unconsummated marriage in an Iranian couple

**DOI:** 10.4314/ahs.v17i3.5

**Published:** 2017-09

**Authors:** Mahshid Bokaie, Zahra Bostani Khalesi, Seyed Mojtaba Yasini-Ardekani

**Affiliations:** 1 Research Center for Nursing and Midwifery Care, Shahid Sadoughi University of Medical Sciences, Yazd, Iran; 2 Department of Midwifery, School of Nursing and Midwifery, Guilan University of Medical Sciences, Rasht, Iran; 3 Addiction and Behavioral Sciences Research Center, Research and Clinical Center for Infertility, Shahid Sadoughi University of Medical Sciences, Yazd, Iran

**Keywords:** Unconsummated marriage, couple's therapy, vaginismus, behavioral therapy

## Abstract

**Background:**

Unconsummated marriage is a problem among couples who would not be able to perform natural sexual intercourse and vaginal penetration. This disorder is more common in developing countries and sometimes couples would come up with non-technical and non-scientific methods to overcome their problem. Multi-dimensional approach and narrative exposure therapy used in this case.

**Methods:**

This study would report a case of unconsummated marriage between a couple after 6 years. The main problem of this couple was vaginismus and post-traumatic stress.

**Results:**

Treatment with multi-dimensional approach for this couple included methods like narrative exposure therapy, educating the anatomy of female and male reproductive system, correcting misconceptions, educating foreplay, educating body exploring and non-sexual and sexual massage and penetrating the vagina first by women finger and then men's after relaxation. The entire stages of the treatment lasted for four sessions and at the one-month follow-up couple's satisfaction was desirable.

**Conclusion:**

Unconsummated marriage is one of the main sexual problems; it is more common in developing countries than developed countries and cultural factors are effective on intensifying this disorder. The use of multi-dimensional approach in this study led to expedite diagnosis and treatment of vaginismus.

## Introduction

Usually, after marriage, intercourse and sexual activity is naturally and mutually expected in both the spouses^1^. Unconsummated marriage means that couple never have sexual relationship^2^. But studies have shown that sometimes not only the intercourse cannot be consummated at the first try, but it could also be delayed for years and even would never happen; this is called unconsummated marriage. Different physical, mental, social and cultural factors are effective in occurrence of this disorder, so it should be considered multi-dimensionally. Unconsummated marriage mainly is due to vaginismus in the woman or premature ejaculation in the man^3^–^6^.

Chakrabarti reported a case of unconsummated marriage after 22 years^7^. The prevalence of this disorder was 8.4% in the study of Barden 8, but considering the role of social and cultural factors in this disorder, the differences in its prevalence between different societies is not unexpected. This disorder is more likely in traditional communities than the modern ones. Different studies have reported its prevalence from 7% to 63.9% in different societies^9^–^11^. Since most of the suffering couples would not seek professional help due to their sense of guilt and shame, guiding them to specialized counseling centers as soon as possible seems necessary^6^. An interesting point is that the main reasons for this disorder are similar in different countries. Some of the reasons for occurrence of unconsummated marriage are lack of sufficient sexual information, sexual performance anxiety, vaginismus, lack of privacy, history of sexual abuse, unreal fears, cultural and social factors, strict religious rules, sexual limitations applied by the family and the society and also specific costumes about the first sexual intercourse, premature ejaculation and erectile dysfunction^2^,^6^,^11^–^15^.

Vaginismus is one of the common sexual disorders among women which is also considered one of the main reasons for unconsummated marriage. The main characteristic of this disorder is involuntary vaginal muscle spasm in women which would make vaginal penetration impossible. This reaction is not just limited to sexual intercourse and the patients would even experience this condition during gynecological examination^16^. Also, the history of sexual abuse is associated with Posttraumatic stress disorder (PTSD), which could lead to this disorder. Unconsummated marriages have different consequences including the sense of guilt, the sense of shame and incompetency, reduced self-esteem, aggression and the sense of frustration, instability in marriage, infertility and divorce^2^,^7^, ^8^,^11^,^12^,^16^,^17^.

Life experiences vary in different people and some women's body would react to sexual activity by vaginismus. Hasty intercourse at the wedding night and its painful experience, unawareness of the anatomy and performance of women's reproductive system and also unawareness of different methods of sexual activity are some of the main reasons for this disorder. Since PTSD should also be considered, narrative exposure therapy was used.

More previous articles are quantitative or prevalence studies. They have used mono-dimensional approach. While recommending the use of multi-dimensional approach in reference books, There is little article detailing this approach it may be useful for young therapists, especially in traditional societies.

## Couple's evaluation

This case with the sense of guilt, shame and incompetency referred to our clinic. The wife was 23 and the husband was 25 years old and they were married for 6 years. Male and female each had a diploma and both of them have a normal weight and height. They were in a moderate financial situation and middle social class. Both of them believed a woman must be virgin until marriage.

The wife was a housewife and the husband was a freelancer. Their marriage was traditional and they were not relatives. Although they had a private bedroom, and nobody living with them, they were not successful in vaginal penetration yet. They were intimate with together. Because they were under pressure of family and local customs, they hid their problem from both families. Man visited a urologist and premature ejaculation and erectile disorder were ruled out. They denied wanting a baby due to family pressure and had referred to a few persons who were not specialists.

Suggested treatments included tying up woman's hands and feet and forceful penetration, using sleeping pills by the woman and then the man tries to penetrate by the man and using narcotics by the woman to reduce pain. Some people had encouraged them to watch pornography videos which decreased woman's sexual desires. A specialist physician recommended them to perform hymenectomy. Although they were very disappointed, they were introduced from one of the health centers to this clinic. They tired from this incomplete sexual relationship. Hymenectomy was not performed because she referred to our clinic before doing this procedure.

At the first session of counseling, the couple seemed disappointed and exhausted about not getting any results from all the treatment methods. They had no problem in their daily life and had no communicational problems. The wife's voice was trembling and her sex drive had reduced recently. The husband's sexual response cycle was complete and he would reach orgasm without vaginal penetration. The wife's evaluation showed normal sex drive and having sexual imagination and desire for sexual activity and sometime, due to clitoral stimulation, she had even reached an orgasm. To havea more comprehensive psychological evaluation of the wife, her history from childhood till now was obtained. After creating an effective communication with the patient, the wife mentioned that during her period of engagement she has witnessed a man raping her sister. After this incidence she had an unpleasant feeling toward touching her body especially her genitals and starting sexual activity would reminded her of the rape scene.

## Treatment method

The main therapist has a PhD in sexual & reproductive health and had different approaches to sexuality. This is the first governmental sex counseling clinic in Yazd/IRAN.

Multi-dimensional approach was used for treating this couple. No pathologic problem was observed in the woman during gynecological examination but she did not allow a full vaginal examination; the primary diagnosis was vaginismus.

Narrative exposure therapy is a method which is used for treating PTSD^18^. Usually people, who have had bitter and horrible experiences in their lives, would try to eliminate those experiences from their minds and forget about them as soon as possible. Affected people believe that the more they run from their experiences and their memories the safer they would be and would have more peace; but this is not true. On the contrary, the more they would escape from their experiences and memories, those memories and experiences would attach to them with more power^19^. Psychological researches have shown that one of the effective methods for reducing the symptoms of PTSD is exposure therapy^20^. In this case dark and frightening events were assessed in detail; the patient was asked to express all of her feelings, physical reactions, voices and thoughts that she had experienced during that incidence during the therapeutic session to her therapist in detail. Then she was asked to write down her memories with details on a paper and then tear it. The purpose of this action was to normalize the emotional responses to fear and create an integrated and meaningful storyline of the patient's life, helping her to reevaluate her negative thoughts and cognitive distortions and coordinating her thoughts and beliefs with her current life conditions. [A16] Multi-dimensional approach was used for treating this couple. This team included someone with a PhD in sexual and reproductive health, aurologist and a psychologist.

## Discussion

Unconsummated marriage is one of the most important sexual disorder more common in developing countries than developed countries, and cultural factors are effective on intensifying it^2^. If not diagnosed, it could be confused with voluntary non-obedience of women and could lead to court orders that would legally and religiously annul the marriage^21^. The presence of the couple together at the counseling center for treatment is necessary and husbands have an essential role in the treatment of this disorder. In most cases husbands are the ones who can help their wives. They should know that, according to the scientific and religious references, this disorder is not a reason for women's non-obedience; these contractions would happen involuntarily and women have no control over them and after a period of treatment, with support from their husbands, women could easily enjoy sexual activities with their husbands^22^. We explained that vaginism is the result of an involuntary vaginal muscle spasm and men should never threaten their wives with divorce because of this disorder. These threats could intensify the muscle spasms and sometimes even lead to sexual desire disorder or sexual aversion in women, which is way more difficult and complicated to treat than vaginismus.

Participation of the man in counseling sessions, non sensate focus and sensate focus which both of them show this problem could treated if both of them are taking part.

This disorder is more common among young women, women with negative attitude towards sexual activity and those who had experienced sexual misconduct or abuse. Barden et al in a study that was conducted on 191 cases of unconsummated marriage in Egypt evaluated its etiologic factors and revealed that the most common factor was psychological disorders, especially performance anxiety, and vaginismus^8^. Abraham 1956 reported 50 cases that were referred to one of the hospitals of London. Some of them were put under surgery with general anesthesia for vaginal dilation, which was useless. He proposed primary gynecology examination and then psychological evaluation. Vaginal dilatation was performed under anesthesia, but the problem was not resolved.

Hamid et al^23^, believed that using the techniques of cognitive-behavioral theory could decrease the anxiety and fear of sexual intercourse in women with vaginismus. This technique may be useful for this couples^24^. Ghorbani et al reported a case of unconsummated marriage after 7 years of marriage. They mentioned unawareness about sexual matters and performance anxiety as the most cause of this disorder. It seems that educating sexual relations as a part of premarital educations is necessary^6^.

Some therapists have expressed duration of treatment was between six months up two years. We were able to treat the patient in four sessions by educating the anatomy of female and male reproductive system, correcting misconceptions, educating foreplay, educating body exploring and sexual and non-sexual massage and narrative therapy. Schover et al. used multi-dimensional approach in their treatment like us.

## Conclusion

Since one of the main goals of marriage is having satisfying marital relations, encountering problems in this dimension could have wide unpleasant effects on all the aspects of individual's life. On the other hand, due to cultural and social limitations, most couples would feel confused when seeking for treatment. Sexual specialized counseling in pre-marital consultations could be helpful and early treatment of this disorder is of great importance in decreasing marital conflicts. Considering the multi-dimensional nature of this disorder, usually specialized treatment of this disorder with help from a team of experts could be effective.
